# Comments on the article “Tissue gadolinium deposition in hepatorenally impaired rats exposed to Gd-EOB-DTPA: evaluation with inductively coupled plasma mass spectrometry (ICP-MS)” by Tomohiro Sato, Tsutomu Tamada, Shigeru Watanabe et al. DOI 10.1007/s11547-014-0492-y

**DOI:** 10.1007/s11547-015-0591-4

**Published:** 2015-11-17

**Authors:** Jan Endrikat, Martin A. Sieber, Jacob Agris, Hubertus Pietsch

**Affiliations:** Global Medical and Clinical Affairs, Radiology, Bayer HealthCare Medical Care, Müllerstrasse 178, 13342 Berlin, Germany; Department of Gynecology, Obstetrics and Reproductive Medicine, University Medical School of Saarland, 66421 Homburg/Saar, Germany; Global Medical and Clinical Affairs, Radiology, Bayer HealthCare Medical Care, 100 Bayer Boulevard, Whippany, 07981 NJ; MR and CT Contrast Media Research, Radiology , Bayer HealthCare Medical Care, Müllerstrasse 178, 13342 Berlin, Germany

To the editor,

We read with great interest the study by Tomohiro Sato et al. published online, January 9, 2015. The authors demonstrated, in an experiment on 2 × 5 rats, that application of 0.625 mmol/kg Gd-EOB-DTPA resulted in significantly higher gadolinium tissue deposition in hepatorenally impaired rats compared to renally impaired rats. The effect was statistically significant in kidneys, spleen and liver but not in other organs.

Based on the methodology used to detect the tissue gadolinium (Gd), the time point of the analysis and the missing validation of the animal model, we think that this experimental setting does not allow any conclusions on gadolinium tissue deposits.

The authors state that “ICP-MS cannot distinguish between chelated and unchelated Gd”. Thus, the time point to analyze Gd tissue deposition is crucial. The authors do not provide a rationale for analyzing tissue 7 days after last GBCA application. At this early time point, it is reasonable to assume that the measurements not only assessed Gd deposits but also chelated soluble Gd. We suggest that the analysis should be done after the majority of this soluble chelated Gd is most likely excreted. Pietsch et al. demonstrated that a steady-state level of skin Gd was reached only after 53 days p.i. of high doses of linear GBCAs. The time to reach steady state depended on the stability level of the linear GBCA [1]. Consequently, we think that 53 days p.i. would be the earliest reasonable time point for determination of Gd tissues deposits.

The excretion kinetics of Gd-EOB-DTPA of the animal model was not thoroughly described prior to start of the experiments. The authors induced hepatic impairment by application of carbon tetrachloride (CCl_4_) without knowing the effect of CCl_4_ on the 1/6 remnant kidney tissue. A reasonable first step should have been to describe Gd-EOB-DTPA kinetics in renally vs hepatorenally impaired animals. If this induction of renal and hepatic impairment results in extremely prolonged excretion kinetics, GBCA would be still circulating 7 day p.i. and would compromise the conclusions on Gd tissue deposits.

In addition, the hepatocyte-specific uptake and hepatobiliary excretion of Gd-EOB-DTPA are based on the saturable uptake by anion-transporting polypeptide 1 transporters (Oatp1) [2]. Liver fibrosis strongly influences the hepatocyte-specific uptake of Gd-EOB-DTPA [3]. Therefore, evaluation of the pharmacokinetics is a pre-requisite to validate this animal model.

Finally, some clinical considerations. Gd-EOB-DTPA was administered 12 times over a period of 6 weeks at a dose of 0.625 mmol/kg. The approved dose of Gd-EOB-DTPA is 0.025 mmol/kg for one single liver MRI, which is usually sufficient for diagnosis. Thus, this 300 times overdosing does not reflect any clinically relevant situation. In the rare case that patients have both severe renal and severe liver impairments, even more caution would be exercised.

In conclusion, based on the methodology used to detect the tissue Gd, the time point of the analysis and the missing validation of the animal model, we think that this experimental setting does not allow any conclusions on gadolinium tissue deposits. The authors only support the broadly accepted hypothesis that reduced elimination kinetics leads to a higher gadolinium exposure.
